# Interaction of TBC1D9B with Mammalian ATG8 Homologues Regulates Autophagic Flux

**DOI:** 10.1038/s41598-018-32003-2

**Published:** 2018-09-10

**Authors:** Yong Liao, Min Li, Xiaoyun Chen, Yu Jiang, Xiao-Ming Yin

**Affiliations:** 1 Department of Pathology, University of Pittsburgh, Pittsburgh, PA USA; 2 Chongqing Medical and Pharmaceutical College, Chongqing, China; 3 School of Pharmaceutical Sciences, Sun Yet-Sen University, Guangzhou Guangdong, China; 4 Department of Pathology and Laboratory Medicine, Indiana University School of Medicine, Indianapolis, IN USA; 5 Department of Chemical Biology and Pharmacology, University of Pittsburgh, Pittsburgh, PA USA

## Abstract

Autophagosomes are double-membraned vesicles with cytosolic components. Their destination is to fuse with the lysosome to degrade the enclosed cargo. However, autophagosomes may be fused with other membrane compartments and possibly misguided by the RAB molecules from these compartments. The mechanisms ensuring the proper trafficking are not well understood. Yeast ATG8 and its mammalian homologues are critically involved in the autophagosome formation and expansion. We hypothesized that they could be also involved in the regulation of autophagosome trafficking. Using the yeast two-hybrid system, we found that TBC1D9B, a GTPase activating protein for RAB11A, interacted with LC3B. TBC1D9B could also interact with other mammalian ATG8 homologues. This interaction was confirmed with purified proteins *in vitro*, and by co-immunoprecipitation *in vivo*. The interacting domain of TBC1D9B with LC3 was further determined, which is unique and different from the known LC3-interacting region previously defined in other LC3-interacting molecules. Functionally, TBC1D9B could be co-localized with LC3B on the autophagosome membranes. Inhibition of TBC1D9B suppressed the turnover of membrane-bound LC3B and the autophagic degradation of long-lived proteins. TBC1D9B can thus positively regulate autophagic flux, possibly through its GTPase activity to inactivate RAB11A, facilitating the proper destination of the autophagosomes to the degradation.

## Introduction

Macroautophagy is referred to as an intracellular degradation activity, which is carried out by the double-membraned autophagosomes^1,2^. Unlike the ubiquitin proteasome system, autophagy is responsible for the degradation of long-lived proteins and intracellular organelles^3^. Autophagy has diverse physiological functions in regulation of energy and nutrient metabolism, in organelle quality control, and in removing misfolded proteins and invading microorganisms^2^. About 41 ATG genes have been defined, which participate in autophagy or autophagy-related process in the yeast^4^, and many have mammalian homologues with highly conserved functions^5^. The core machinery seems to be built around two ubiquitin-like conjugation systems^6^. In one system, the ubiquitin-like protein, ATG12, is first activated by ATG7, an ubiquitin-activating enzyme (E1)-like protein, and then transferred by ATG10, an ubiquitin carrier protein (E2)-like protein, to ATG5. The covalently bound ATG5-ATG12 complex interacts with ATG16 to form a multimer complex, which is localized to membranes of early autophagosomes. In another conjugation system, ATG8, or one of its mammalian homologues such as LC3B, is first cleaved by a cysteine protease, ATG4, to expose the conserved C-terminal glycine. ATG8/LC3B is then conjugated to phosphatidylethanolamine (PE), mediated by the E1-like molecule ATG7, and another E2-like molecule, ATG3^7,8^. The ATG12-ATG5-ATG16 complex that anchors on the autophagosomal membranes can further accelerate the LC3B-PE conjugation^9^.

In yeast, there is only one ATG8 protein, but in mammals, there are seven ATG8 homologues, LC3A-a, LC3A-b, LC3B, LC3C, GABARAP, GATE-16 (GABARAPL2), and ATG8L (GABARAPL1)^10^. Their expressions vary in different tissues, suggesting that there might be tissue-specific importance of these homologues^11–13^. While these proteins possess some non-autophagy functions, they are important to the autophagy process in a number of ways^10^. Much of the knowledge in the mammalian system is from the study of LC3B, which has been widely used as an autophagosome marker^14,15^. Studies have shown that the ATG8 molecules have functions in membrane fusion, which can be crucial to the expansion or elongation of autophagosomes^16^ and autophagosome size^17^. In addition, GABARAP and GABARAPL1 can serve as scaffolding proteins by recruiting ULK1 and Beclin-1 to the nucleation site of autophagosomes^18^. Another important function of ATG8 and its homologues is that they can interact with autophagy adaptor proteins^19^, such as sequestosome 1 (SQSTM1/p62)^20^, optineurin (OPTN)^21^, and NIX/BNIP3L^22^. The adaptor molecules bind to the ATG8 homologues through the LC3-interacting region (LIR), and at the same time bind to cargos molecules, thus facilitating the engulf of cargo molecules by the growing autophagosomes for degradation^6^.

TBC (Tre-2/Bub2/Cdc16) domain is a conserved protein motif found in all eukaryotes, which consists of approximately 200 amino acids^23^. The TBC domain possesses the activity of the GTPase activating protein (GAP) for the small GTPase, RAB^24^. RAB proteins are found on distinct intracellular membranes and regulate a variety of membrane trafficking events including vesicle budding and uncoating, motility, tethering and fusion, through recruitment of different effector molecules^25^. Some of the RAB molecules, such as RAB5^26^, RAB7^27^, RAB24^28^, and RAB33B^29^, have been reported to participate in the biogenesis and maturation of autophagosomes^30^. Interestingly, several TBC-family GAPs have been found to affect autophagy, perhaps through their interaction with the ATG8 family proteins^31–33^.

In this study, we identified a new TBC-family GAP, TBC1D9B, that interacts with LC3B using the yeast two-hybrid system. We identified a new interaction domain in the TBC1D9B, which is distinguished from the common LIR found in other LC3B-interacting molecules. Furthermore we found that TBC1D9B could be colocalized with LC3B on autophagosome membranes and its function was to promote autophagic flux. These results suggest that some of the GAP molecules can be recruited to the autophagosomes via their interaction with LC3 to promote autophagy function, possibly by inactivating the corresponding RAB molecules to prevent mis-trafficking.

## Results

### Identification of TBC1D9B as an interacting protein for ATG8 homologues

**Figure 1:**
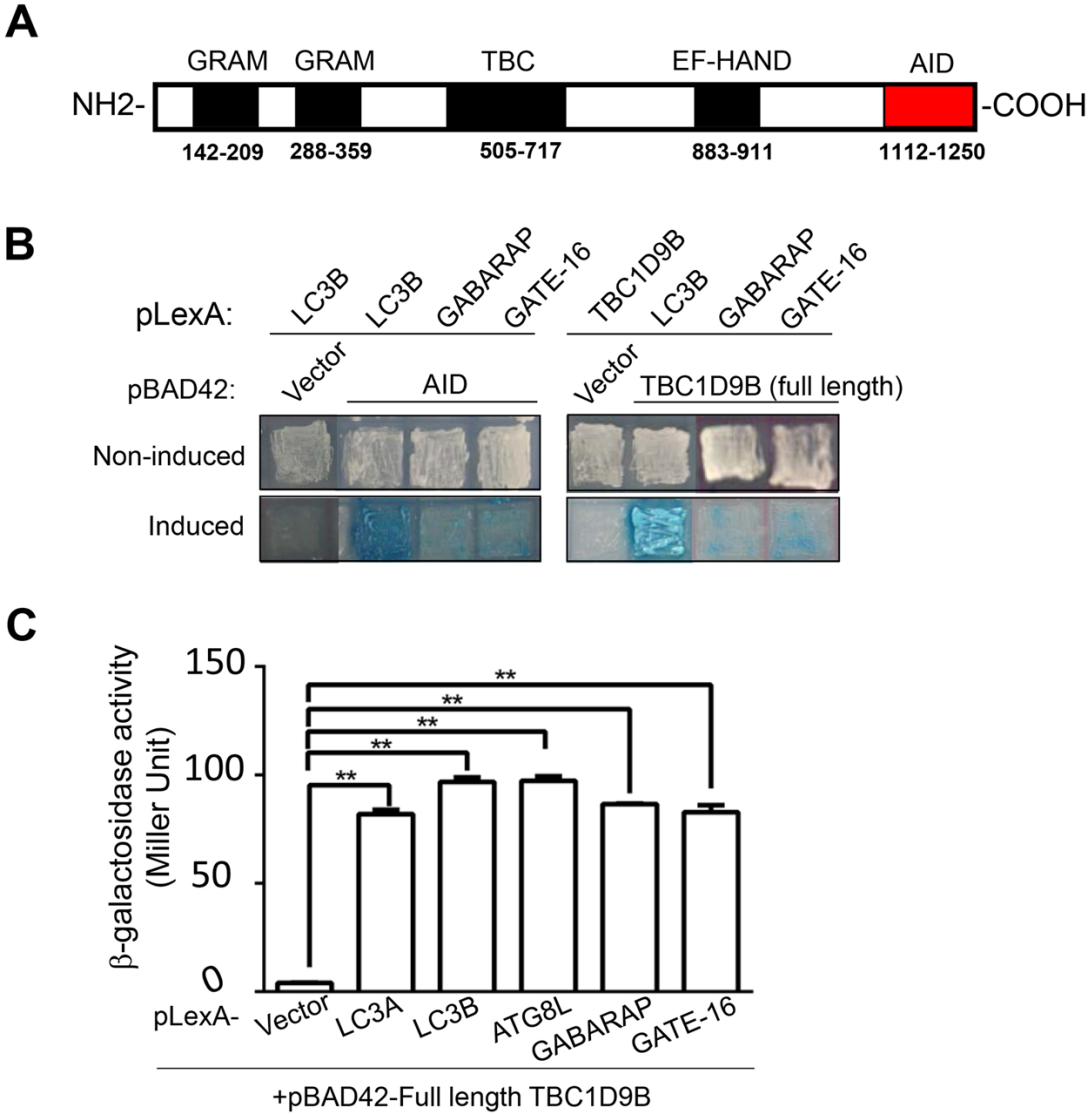
Identification of TBC1D9B as an interacting protein of mammalian ATG8 homologues. (**A**) Schematic representation of human TBC1D9Bα (NM_198868.2, Gene ID: 23061). The locations of the TBC domain and other domains are indicated with the corresponding amino-acid position. The newly defined ATG8-interacting domain (AID) at the C-terminal end is also denoted. (**B**) Human LC3B was used as a bait to screen for potential interacting proteins using the yeast two-hybrid system. A fragment of 140 amino-acid, i.e. the AID of TBC1D9B, was identified. AID and full length TBC1D9B were further examined for the ability to interact with GABARAP and GATE-16. The cells were cultured in non-induced or induced condition as described in the Method section. Photos were taken after 5 days in culture. Positive interactions resulted in blue colonies in induced condition. (**C**) After co-transformation with pBAD42-TBC1D9Bα (full length) and various p-LexA-ATG8 homologues, yeast were placed in fresh liquid culture overnight and the β-galactosidase activity was measured (n = 3 cultures per group). Data shown are mean ± SEM. **p > 0.01.

In order to better understand the function of ATG8 family proteins in autophagy, we screened a cDNA library for interacting proteins of the most commonly studied mammalian ATG8 family molecule, LC3B, using the yeast two-hybrid system. Among the positive hits, one hit had been found repeatedly with a frequency of 45% (38/84). Sequencing confirmed that the hit was the C-terminal fragment of TBC1D9B (amino acids 1112–1250) (Fig. 1A). TBC1D9B belongs to the TBC family of GAP for the RAB proteins^23,24^. It contains two GRAM domains, one TBC domain, and one EF-HAND domain. We named the newly defined LC3B-interacting fragment as the ATG8 interaction domain (AID). We further found that AID also interacted with other ATG8 homologues, GABARAP and GATE-16 (Fig. 1B). As expected, the full-length TBC1D9B was able to interact with ATG8 homologues with a similar potency (Fig. 1B,C).

**Figure 2:**
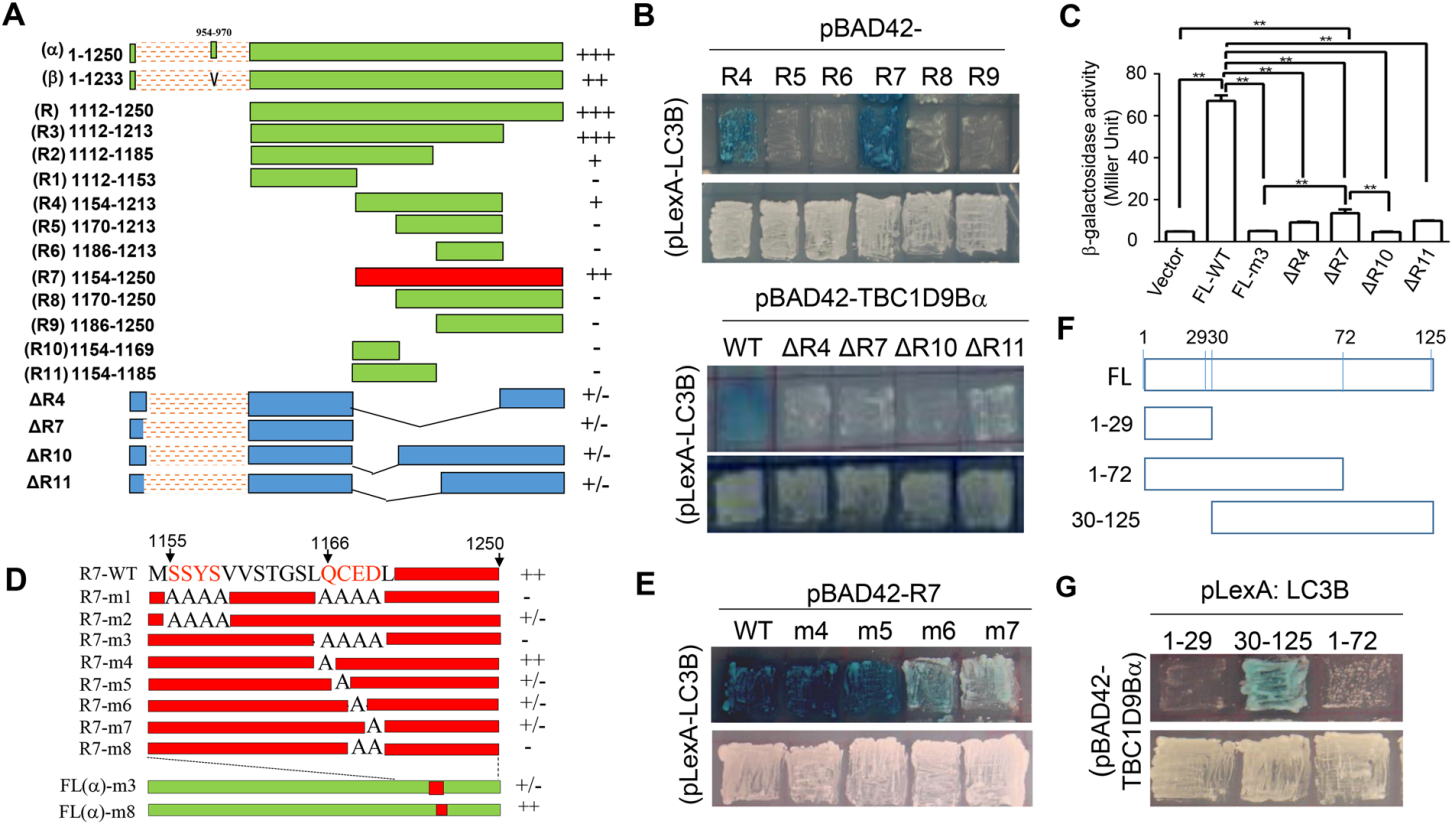
Characterization of key residuals in TBC1D9B for the interaction with LC3B. (**A**) Schematic representation of the full length and truncated human TBC1D9B. TBC1D9B has two splicing variants, the alpha form (NM_198868.2) and the beta form (NM_015043.3). The beta form did not contain the sequence from 954 to 970. The amino acid positions for each TBC1D9Bα fragment (R and R1-R11, in green and red color) as well as truncational mutants of the full length TBC1D9Bα (ΔR4, ΔR7, ΔR10, and ΔR11, in blue color) were indicated. Strength of the interaction (− to +++) with LC3B in the yeast two-hybrid system was indicated next to the bars. Fragment R7 (in red) represents the minimal length of AID that could give rise to the optimal interaction. (**B**) TBC1D9B fragments (upper panel) or truncational mutants (lower panel) cloned on the pBAD42 vector were co-transformed with pLexA-LC3B into the yeast and cultured in the induced condition as described in the Method section. (**C**) After co-transformation with pBAD42-TBC1D9Bα (full length, wild type or the various mutants), and p-LexA-LC3B, yeast cells were placed in fresh liquid culture overnight and the β-galactosidase activity were measured (n = 3 cultures per group). Data shown are mean ± SEM. **p > 0.01. (**D**) Schematic representation of alanine substitution in the R7 fragment of TBC1D9Bα and in the full length TBC1D9Bα. The amino acid positions were indicated. Strength of the interaction (− to +++) with LC3B in the yeast two-hybrid system was indicated next to the bars. (**E**) The R7 fragment of TBC1D9B and its mutated variants were cloned into pBAD42 and co-transformed with pLexA-LC3B into the yeast as in panel B. (**F**) Schematic representation of the full length (FL) and truncated human LC3B. Amino acid positions were indicated. (**G**) LC3B fragments were cloned into pLexA and co-transformed with pBAD42-TBC1D9Bα(FL, wild type) into the yeast and cultured as in panel B. All photomicrographs (panels B, E, G) were taken after 5 days in culture. Strength of interaction shown in panels A and D is based the number of days in culture for the colony to develop the blue color at 30 °C. ^+++^1–2 days, ^++^3 days, ^+^4 days, ^+/−^5–6 days, ^−^more than 6 days.

Sequence analysis of TBC1D9B (Gene ID: 23601) found that this molecule had two splicing variants, the alpha form (NM_198868.2) and the beta form (NM_015043) (Fig. 2A). The beta form did not contain the amino acid sequence from 954 to 971, and thus 17 amino acids shorter than the alpha form. Both forms were able to interact with LC3B as both had the AID, but the alpha form seemed to bind more strongly. To determine whether sequences within AID in the full length TBC1D9B were sufficient for the interaction with LC3B, we conducted deletion mutagenesis of AID and examined the ability of these truncated mutants to interact with LC3B in the yeast two-hybrid system (Fig. 2A–C).

We found that the smallest interacting domain spanned the region from aa 1154 to aa 1213 (R4). A slightly bigger fragment (R7, aa 1154–1250) was able to interact with LC3B as potently as the beta form of the full length TBC1D9B (Fig. 2A,B). Deletion of additional amino acids (R5, R6, R8, R9, R10, R11) resulted in loss of the interaction with LC3B. To determine whether R7 was essential for TBC1D9B-LC3B interaction we deleted R7 or a part of R7, *i*.*e*., R4, R10, and R11, from the full length TBC1D9Bα. We found that these truncational mutants were not able to interact with LC3B (Fig. 2A–C). These studies indicated that AID or its minimal sequence (R4, aa 1154–1213) was necessary and sufficient for TBC1D9B to interact with LC3B.

### Interaction of TBC1D9B with LC3B depended on a new LC3 binding motif

Previously identified LIR in adaptor molecules such as p62/SQSTM1 contains the signature WxxL/I motif ^20,34^. Two WxxL motifs and one WxxI motif were found in TBC1D9B at the position of aa 87–90, aa 174–177 and aa 199–202. However, the WxxL/I motif was not found in AID (aa 1112–1250). Thus WxxL motifs could be dispensed for the interaction of TBC1D9B with LC3 and other ATG8 homologues. The results also indicated that TBC1D9B-LC3 interaction could be mediated by other critical amino acids. To determine the key amino acids involved in the interaction, we conducted site-directed mutagenesis of the R7 fragment and examined the ability of the mutants in the yeast two-hybrid system. We identified two clusters of amino acids (S^1155^S^1156^Y^1157^S^1158^ and Q^1166^C^1167^E^1168^D^1169^) in the R7 region to be important in interacting with LC3B (Fig. 2C–E). Alanine substitution of all eight amino acids (m1 mutant), or the four amino acids in one of the two clusters (m2 or m3 mutant) significantly reduced or completely eliminated the interaction. We examined the Q^1166^C^1167^E^1168^D^1169^ cluster in more details by single amino acid mutation and found that C^1167^ or E^1168^ or D^1169^ (m5, m6, m7 mutants, respectively) was more critical than Q^1166^ (m4 mutant) (Fig. 2D).

Interestingly, while alanine replacement of E^1168^D^1169^ (R7-m8) was sufficient to disable the ability of R7 to interact with LC3B, it only slightly weakened the ability of the full length TBC1D9Bα to interact with LC3B, while alanine replacement of all four amino acids (Q^1166^C^1167^E^1168^D^1169^, R7-m3) also had retained some ability to interact with LC3B (Fig. 2D). These findings suggested that other amino acids in the R7 domain could contribute to the interaction when this domain was properly positioned in the full length molecule.

The structure of LC3B is clearly divided into the N-terminal part (aa 1–29) and the C-terminal part (aa 30–125) (Fig. 2F) based on crystalized structure (PDB ID: 3WAL). The N-terminal part was not able to interact with TBC19D9B, whereas the C-terminal part was (Fig. 2G). Since the fragment with amino acids 1–72 could not interact with TBC1D9B, the binding might mostly rely on the sequence from amino acids 72 to 125.

### TBC1D9B directly interacted with LC3 *in vitro* and in mammalian cells

**Figure 3:**
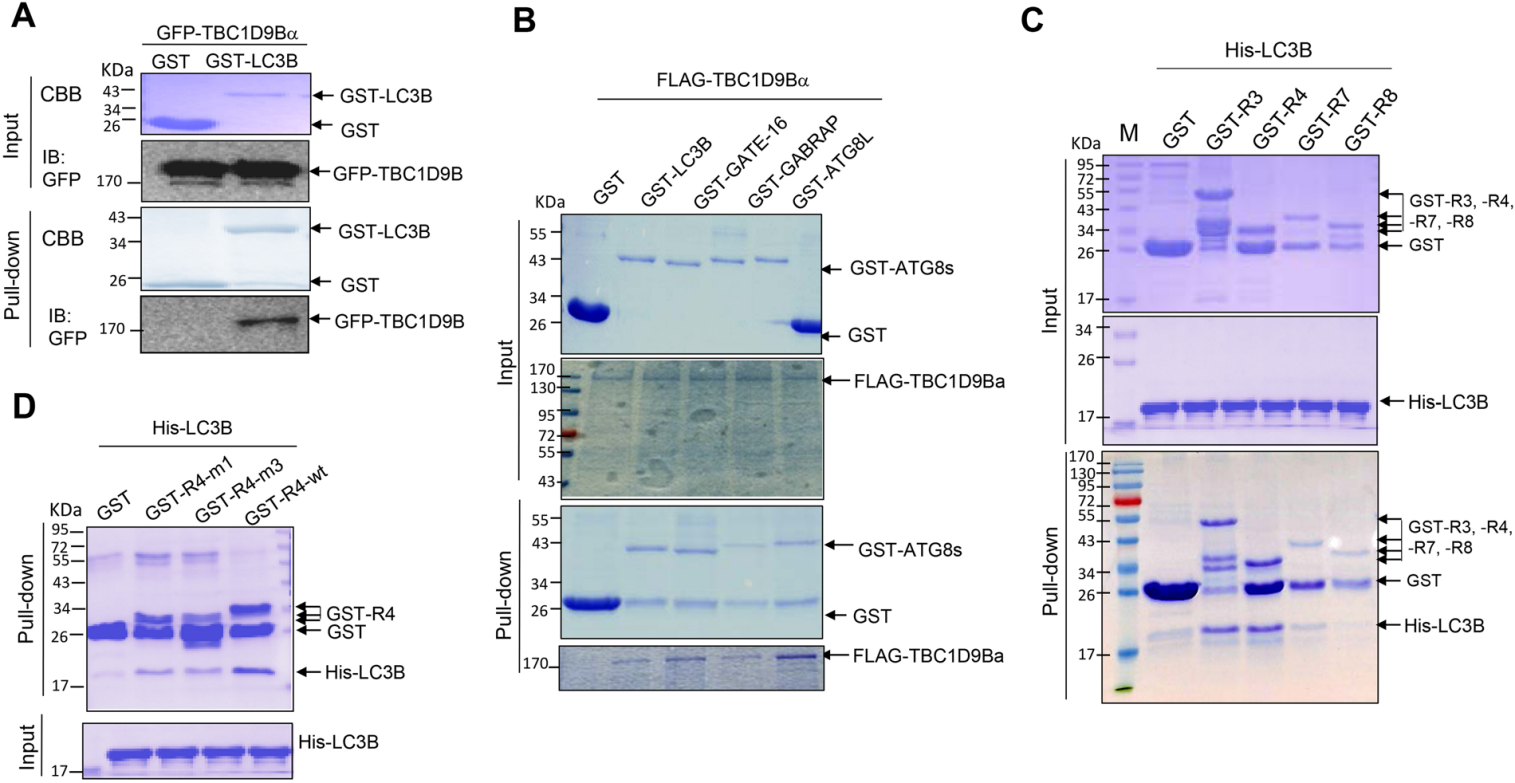
Mammalian ATG8 homologues interacted with TBC1D9B *in vitro*. (**A**) Recombinant GST or GST-LC3B proteins were mixed with lysates of HEK293 cells stably expressing GFP-TBC1D9Bα. (**B**) Recombinant GST or GST-fused mammalian ATG8 homologues were mixed with recombinant FLAG-TBC1D9Bα. (**C**,**D**) Recombinant GST or GST-fused fragments of TBC1D9Bα were mixed with recombinant His-LC3B. For the pull-down assay, glutathione beads were then added to pull down GST or GST-fused proteins and the interacting molecules. The pull-down materials were resolved on 8 or 12.5% SDS-PAGE, followed by staining with CBB (**A–D**) or by immunoblotting with anti-GFP. (**A**) The presence of GST in some of the GST-fusion groups was due to instability of the recombinant proteins.

To determine whether TBC1D9B could directly interact with LC3B without the involvement of other factors, we first used recombinant GST-fused LC3B proteins to examine its ability to pull down GFP-TBC1D9B expressed in HEK293 cells. The fusion protein was mixed with HEK293 lysates and was indeed able to interact with TBC1D9B in this pulldown assay (Fig. 3A). We then prepared FLAG-tagged recombinant TBC1D9B and GST-fused ATG8 homologues for the *in vitro* GST pulldown assay. GST-fused LC3B, GATE-16, GABARAP and ATG8L could clearly interact with FLAG-tagged TBC1D9B in this assay (Fig. 3B), confirming the results from the yeast two-hybrid assay (Fig. 1B) and indicating that the ATG8 homologues could directly interact with TBC1D9B.

Conversely, GST-fused recombinant TBC1D9B fragment, R3, R4 or R7 was able to pull down His-tagged recombinant LC3B, whereas GST-fused R8 fragment was not able to do so (Fig. 3C), confirming the yeast two-hybrid results (Fig. 2D). GST-fused R4 fragment with m1 or m3 mutation (Fig. 2D) had a significantly reduced ability to interact with His-tagged LC3B (Fig. 3D). Although it seems that protein interactions determined by the GST pulldown assay was less sensitive than that determined by the yeast two-hybrid assay for mutations at the sites of SSYS (1155–1158) and QCED (1166–1169), the two assays collectively indicated the importance of these amino acids in the interaction of TBC1D9B with LC3B.

**Figure 4:**
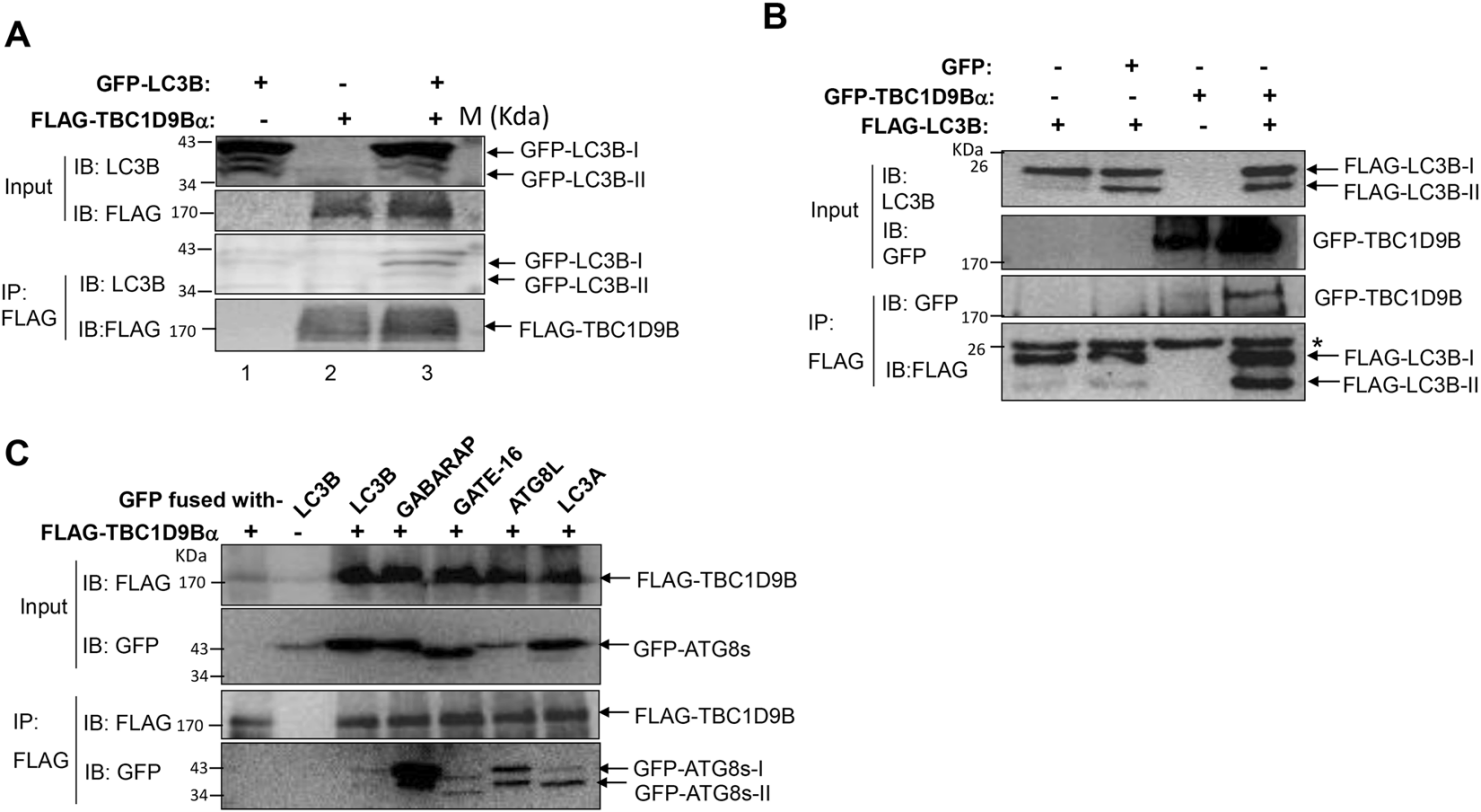
Interaction of mammalian ATG8 homologues with TBC1D9B *in vivo*. (**A**) HCT116 cells stably expressing GFP-LC3B (lane 1, 3) or none (lane 2) were transfected with FLAG-TBCD9Bα (lane 2, 3) for 24 hours. Cell lysates were subjected to immunoprecipitation with anti-FLAG antibody to pull down TBC1D9Bα. The precipitates were then analyzed by immunoblotting using anti-LC3B antibody for the presence of LC3B, and anti-FLAG for the presence of TBC1D9Bα. (**B**) HEK 293 cells were transfected with FLAG-LC3B, together with GFP-TBC1D9Bα or GFP as indicated for 24 hours. Cell lysates were subjected to immunoprecipitation with anti-FLAG antibody to pull down LC3B. The precipitates were then analyzed by immunoblotting with an anti-FLAG antibody for the presence of LC3B, and anti-GFP for the presence of TBC1D9Bα. (**C**) HEK293 cells stably expressing FLAG-TBC1D9Bα were transfected with various GFP-fused mammalian ATG8 homologues for 24 hours. Cell lysates were subjected to immunoprecipitation with anti-FLAG antibody to pull down TBC1D9B. The precipitates were then analyzed by immunoblotting using anti-GFP antibody for the presence of ATG8 homologues, and anti-FLAG for the presence of TBC1D9Bα. The input shown was 1–2% of total used in immunoprecipitation assay. *Non-specific bands.

To evaluate the interaction of TBC1D9B and LC3B in mammalian cells, we performed co-immunoprecipitation assay. HCT116 cells stably expressing GFP-LC3B or control cells were transfected with or without FLAG-TBC1D9B for 24 hours. The cell lysate was subjected to co-immunoprecipitated with anti-FLAG, followed by immunoblotting assay with anti-LC3B (Fig. 4A). Conversely, HEK293 cells stably expressing FLAG-LC3B or the control cells were transfected with GFP-TBC1D9B, or the GFP vector for 24 hours. The cell lysate was subjected to co-immunoprecipitation with anti-FLAG, followed by immunoblotting with anti-GFP (Fig. 4B). In both cases, the *in vivo* interaction of the two molecules was detected. *In vivo* interaction of FLAG-TBC1D9B with several other ATG8 homologues (GABARAP, GATE-16, ATG8L, and LC3A) in HEK293 cells were also confirmed by co-immunoprecipitation assay (Fig. 4C). Taken together, TBC1D9B interacted with the ATG8 homologues *in vitro* and *in vivo*, in the yeast cells and in the mammalian cells.

### TBC1D9B promoted autophagic flux

**Figure 5:**
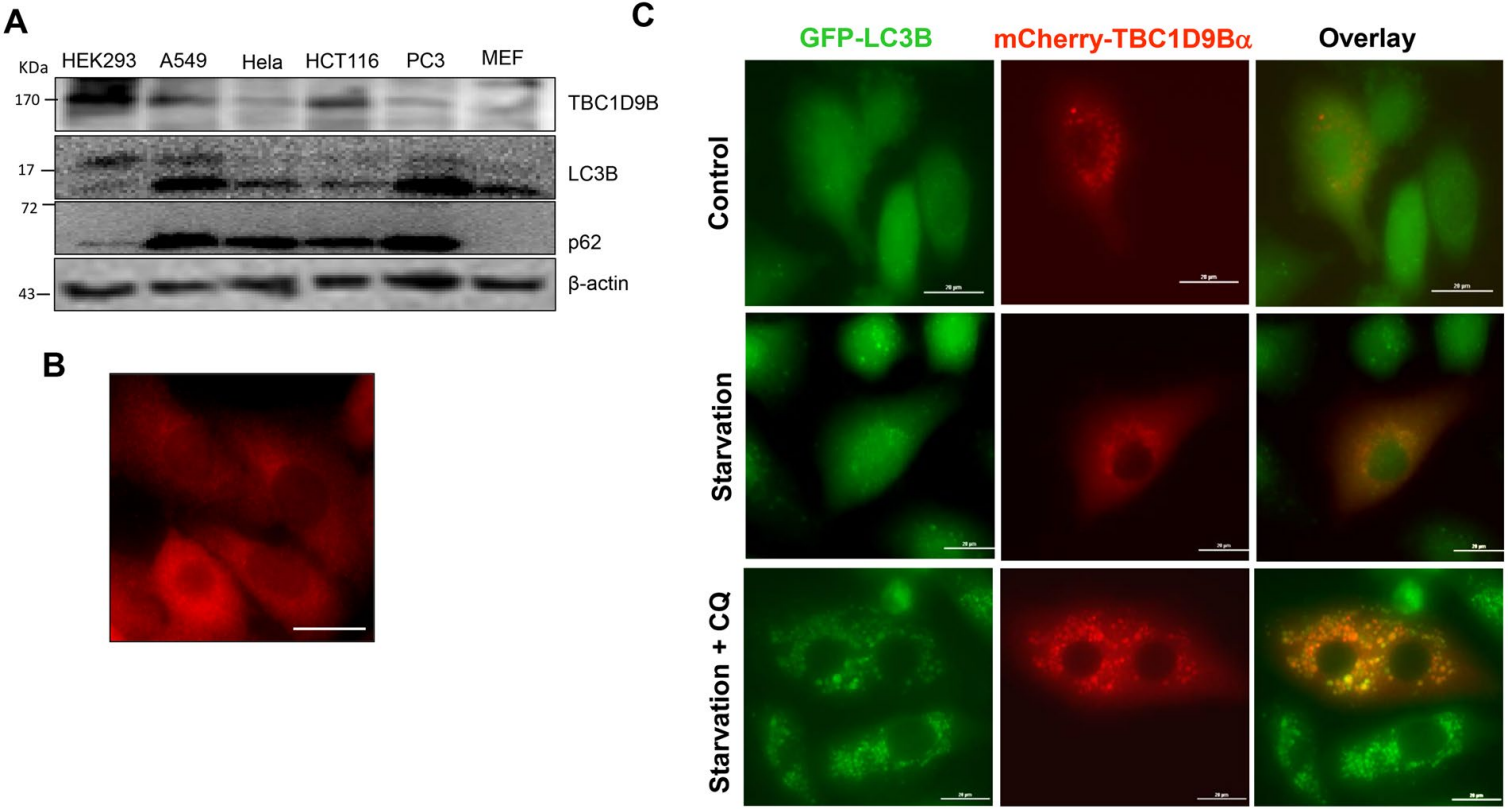
Colocalization of TBC1D9B with LC3B in starved cells. (**A**) Cell lysates (40 µg) prepared from the indicated cell lines were subjected to immunoblotting assay using a rabbit polyclonal anti-human TBC1D9B antibody. (**B**) A549 cells were fixed and stained with the rabbit anti-TBC1D9B antibody followed by Cy-3-labelled goat anti-Rabbit secondary antibody. TBC1D9B is expressed mainly in the cytosol with a recognizable concentration in the peri-nuclear region. (**C**) A549 cells stably expressing GFP-LC3B were transfected with mCherry-TBC1D9Bα for 24 hours and then cultured in normal complete medium supplemented with 10% serum (control), in EBSS with no amino acid and serum (starvation), or in EBSS plus chloroquine (CQ, 10 μM), for 3 hours. Colocalization of TBC1D9B with the LC3B puncta could be observed in starvation condition. Scale bar: 20 µm.

Several mammalian cell lines, including HEK293, A549, HCT116, Hela, PC3 and mouse embryonic fibroblasts (MEF) express endogenous TBC1D9B to different degrees, which, however, did not seem to be correlated with the level of LC3B or p62/SQSTM1 (Fig. 5A). Immunostaining showed that endogenous TBC1D9B was diffusively expressed in the cytosol with a recognizable concentration in the peri-nuclear region (Fig. 5B). In A549 cells stably expressing GFP-LC3B transfection of mCherry-TBC1D9Bα resulted in a similar cellular distribution in the normal culture condition with complete medium supplemented with 10% serum (Fig. 5C). However, when such cells were cultured in serum-free EBSS with no amino acids GFP-LC3 puncta formed, indicating the initiation of autophagy in the starvation condition (Fig. 5C). TBC1D9Bα was mildly concentrated with LC3B puncta, which became more prominent when a lysosome inhibitor, chloroquine (CQ), was added to the EBSS, blocking the degradation of autolysosomes. The immunostaining assay demonstrated that TBC1D9B could colocalize with LC3B, likely through their interaction, following autophagy stimulation.

**Figure 6:**
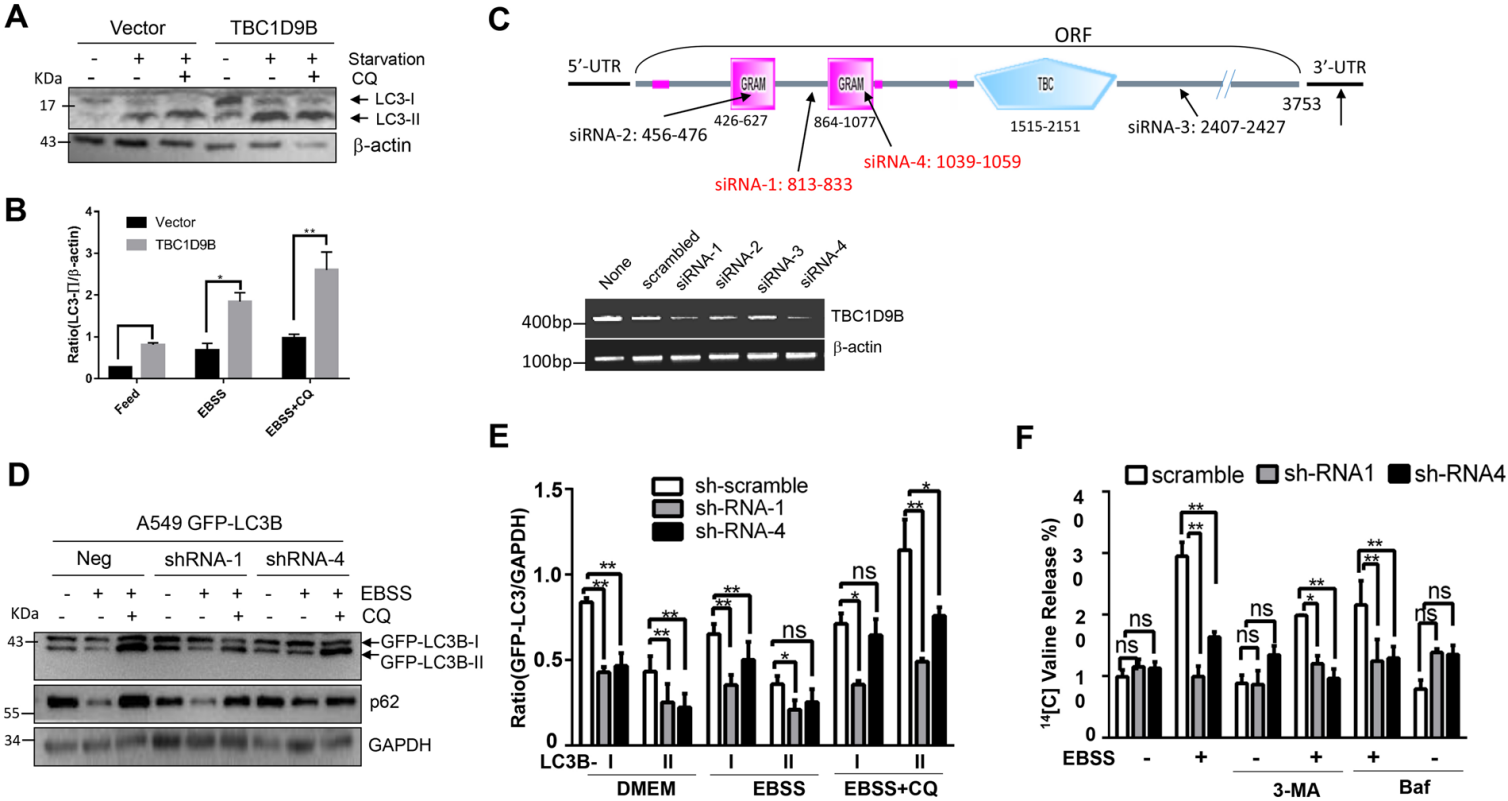
TBC1D9B can promote autophagy. (**A**,**B**) HEK293 cells stably expressing TBC1D9Bα or the vector control were cultured in complete medium or EBSS with or without CQ (10 μM) for 5 hours. Immunoblotting analysis was conducted with anti-LC3 and anti-β-actin (**A**) The level of lipidated form of LC3 (LC3-II) was compared after normalization with the loading control by densitometry (**B**). (**C**) Location of the four TBC1D9Bα-targeting siRNAs used in this study. A549 cells were transfected for 72 hours with a scrambled or specific siRNA against TBC1D9Bα as indicated. RT-PCR was performed for the expression of TBC1D9B. siRNA-1 and siRNA-4 were most effective. (**D**,**E**) A549 cells stably expressing GFP-LC3B were transduced with negative or specific lentivirus carrying a shRNA against TBC1D9B as indicated for 72 hours. Cells were subsequently treated as indicated with or without CQ (10 μM), and analyzed by immunoblotting assay. (**D**) The levels of GFP-LC3-I or GFP-LC3-II were quantified by densitometry (**E**). (**F**) A549 cells stably expressing a scrambled shRNA or TBC1D9Bα-specific shRNA were treated with complete medium (DMEM), or EBSS with or without 3-MA (10 mM) or Bafilomycin A1 (50 nM) for 5 hours. Long-lived protein degradation was then determined. Data shown are mean ± SEM. *p < 0.05; **p < 0.01. n.s.: no significance.

To determine the functional role of TBC1D9B, we examined the impact of this molecule on autophagic flux, based on the turnover of LC3B-II, the membrane form of LC3B. HEK293 cells stably expressing vector or TBC1D9Bα were cultured in complete or starvation medium (EBSS) with or without the lysosome inhibitor, chloroquine (CQ) for 5 hours. The level of endogenous LC3B-II was determined by immunoblotting. We found that overexpression of TBC1D9B increased the level of LC3-II, which was further elevated by the co-treatment of CQ (Fig. 6A,B), suggesting that TBC1D9B could promote autophagic flux.

To inhibit the expression of endogenous TBC1D9B, several specific siRNAs were designed. siRNA-1 and siRNA-4 were found to be most effective (Fig. 6C). Two specific shRNA vectors were constructed based on the siRNA-1 and siRNA-4 sequences, respectively and introduced to A549 cells stably expressing GFP-LC3 (Fig. 6D,E). Culture in EBSS medium triggered autophagic degradation, resulting in a lower level of GFP-LC3 due to degradation, which was reversed with the inclusion of the lysosome inhibitor CQ in the medium. The level of the autophagy substrate p62 altered in a way consistent with the change in degradation capability. When cells were treated with siRNA-1 or siRNA-4 against TBC1D9B, the magnitude of the change in the level of GFP-LC3 or the level of p62 was reduced, indicating that autophagic degradation was reduced by the knockdown of TBC1D9B.

To determine whether functionally TBC1C9B promoted autophagy, we analyzed the degree of long-lived protein degradation under starvation condition. This is a reliable functional assay for autophagy in mammalian cells^35^, which is activated by starvation and suppressed by the class III PI-3 kinase inhibitor, 3-MA, and the lysosomal inhibitor, Bafilomycin A1(Fig. 6F). We found that long-lived protein degradation was significantly reduced when the expression of TBC1D9Bα was significantly inhibited by the effective shRNA (Fig. 6F). Taken together, TBC1D9B could promote autophagy, likely as the result of being recruited to the autophagosome via its interaction with LC3B.

## Discussion

This study had defined TBC1D9B, a GAP for RAB11A^36^, as a binding protein of the mammalian ATG8 homologues. TBC1D9B can functionally promote autophagic flux based on the effect on the LC3-II turnover and the long-lived protein degradation.

### TBC1D9B can promote autophagy via the interaction with ATG8 homologues

TBC1D9B can interact with multiple mammalian ATG8 homologues, including LC3A, LC3B, GATE-16, GABARAP and ATG8L. In the present study the interaction was initially discovered through the yeast two-hybrid system, but it has been confirmed *in vitro* with recombinant proteins and in mammalian cells through co-immunoprecipitation assay. Other investigators, by screening a battery of TBC family proteins, had also identified TBC1D9B as a potential LC3-interacting protein using similar methodologies^31^. The strength of the interaction of TBC1D9B with the different mammalian homologues seems different based on the beta-galactosidase assay. However, the actual difference in binding affinity needs to be assessed by a more defined measurement, such as that based on surface plasmon resonance.

The yeast ATG8 and its mammalian homologues are known to interact with a number of adaptor molecules, which are important for the recruitment of cargos to the autophagosome for degradation^19^. These adaptor molecules, including p62/SQSTM1^20^, optineurin (OPTN)^37^ and NIX/BNIP3L^38^, contain the LC3-interacting region (LIR) with the signature WxxL/I motif ^20,34^. Some TBC family molecules, such as TBC1D5, could interact with the ATG8 homologues via the LIR motif ^31^. But having LIR motif alone is not sufficient to interact with ATG8 homologues because almost all TBC proteins have the LIR motif, but not all of them can interact with LC3^31^. In addition, LIR may be dispensable for the interaction as shown in the present study with TBC1D9B.

The required domain for TBC1D9B to interact with LC3B, as initially defined by the yeast two-hybrid screening, was a C-terminal fragment of 139 amino acids, termed as AID. AID can be further trimmed into a fragment of 97 amino acids (R7, aa1154–1250) that retains the most binding capability (Fig. 2A,B). AID or the R7 region does not contain the WxxL motif. Instead, we have found that two clusters of amino acids (S^1155^S^1156^Y^1157^S^1158^ and Q^1166^C^1167^E^1168^D^1169^) are required for the interaction with LC3B. Alanine substitution of either of the cluster was sufficient to disable the binding. Interestingly, the SSYS motif can also be found in TBC1D4 and TBC1D5, whereas an xCED (x is any amino acid) motif is found in TBC1D1 and TBC1D4. These two new binding motifs (SSYS and xCED) in the TBC family proteins could be important for interacting with the ATG8 homologues.

This study has also found that there are two splicing variants of TBC1D9B, the α form and the β form. The β form lacks the sequence from aa 964 to aa 970, compared to the α form (Fig. 2A). The β form is anticipated to have similar GAP activity or function as all the functional domains are retained. In addition, the SSYS and QCED motifs are present in the β form, consistent with its ability to interact with LC3B (Fig. 2A).

In this study TBC1D9B was found to promote autophagic flux under the normal expression level since a knockdown of TBC1D9B inhibited the LC3-II turnover and the autophagic degradation of long-lived protein in response to starvation. Conversely, over-expression of TBD1D9B promoted LC3-II turnover. Notably, an earlier study found that overexpression of TBC1D9B inhibited starvation-induced autophagy in 2GL9 cells based on the level of GFP-LC3-II^39^. This discrepancy may be due to the different type of cells being used or the lack of confirmatory studies with the knockdown approach in the previous work. Overall, we would favor that TBC1D9B functions to promote autophagy in general.

It is interesting to note that among the 40 different TBC domain-containing GAP^40^, fifteen have been found to be able to interact with ATG8 homologues, and nine have been found to affect autophagy functionally^31–33^. Other than TBC1D9B, TBC1D20 could also maintain autophagic flux at the normal expression level^41^. A defective TBC1D20 function results in eye, testicular and neuronal abnormalities and the failure in the removal of damaged proteins and organelles in lens fiber cells^41^. But TBC1D2^42,43^, TBC1D14^39,44^ and TBC1D20^41^ seemed to be able to inhibit autophagy. Thus TBC-family proteins may have different effects on autophagy.

### TBC1D9B may regulate autophagosome maturation by preventing mis-trafficking

As a GTPase activating protein, the TBC family molecules promote the transition of GTP to GDP bound to the corresponding RAB molecules, thus inactivating their functions. There are more than 40 different TBC proteins in mammalian, and each of them can catalyzes a specific RAB protein^40^. Interestingly, many of the RAB molecules, such as RAB5^26^, RAB7^27^, RAB24^28^ and RAB33B^29^, have been found to participate in autophagy in mammalian cells^30^, which can thus be affected by TBC family of GAPs.

More relevant to this study, RAB11 has been shown to regulate autophagy^39,45^. In one study of mammalian cells, RAB11 was found to be required for autophagosome formation through the contribution of the recycling endosome to autphagosome^39^. In another study with *D*. *melanogaster*, RAB11 was found to promote later endosomes to fuse with autophagosomes to support autophagic flux^45^. In our previous works, we identified TBC1D9B as a GAP protein to RAB11A^36^. TBC1D9B could thus affect autophagy in a way related to RAB11.

**Figure 7:**
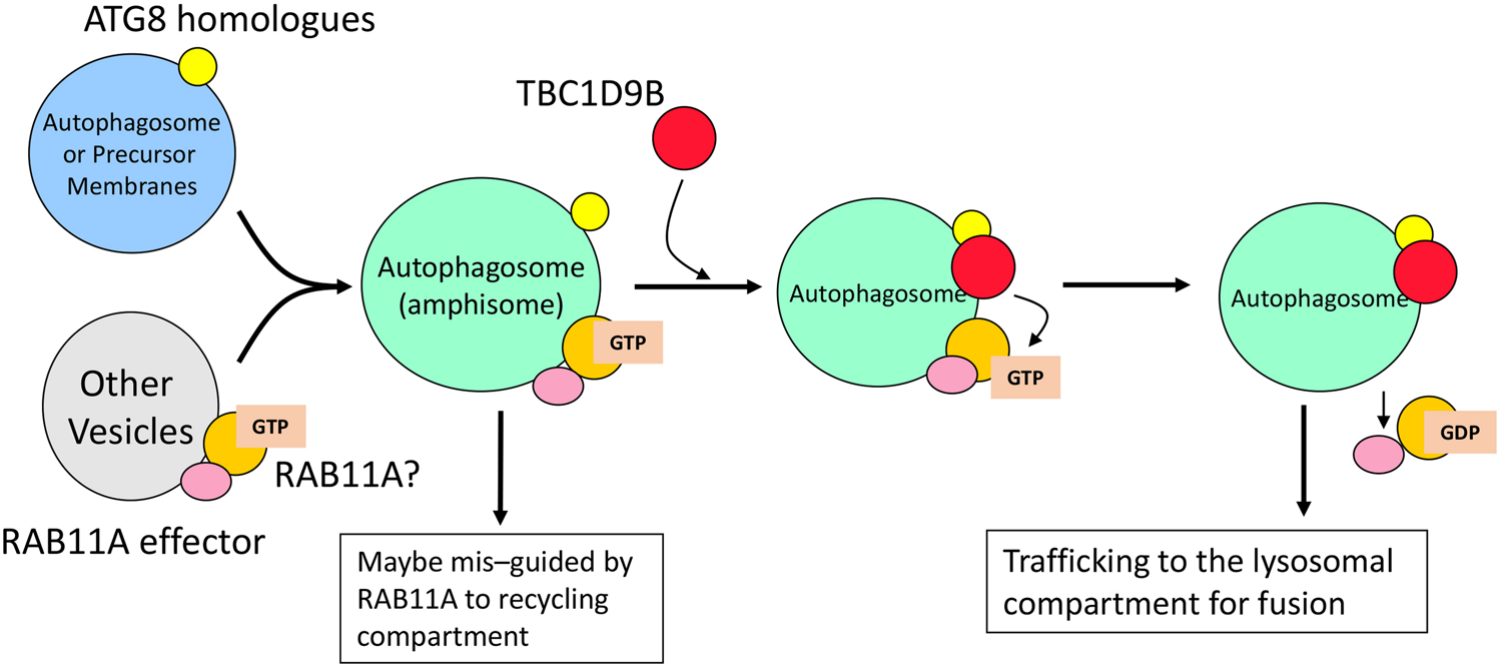
A model for the role of TBC1D9B in autophagy. Multiple fusion of autophagosomes (blue) with other types of vesicles (gray) can occur during the biogenesis/maturation of autophagosomes in mammalian cells. The resultant vesicles, also known as amphisomes (green), could retain RAB molecules that are specific to the donor vesicles. Continuous presence of these RAB molecules may “misguide” the autophagosome to the trafficking pathway specified by these RAB molecules (orange) and their associated effector molecules (pink) and thus negatively affect autophagosome trafficking to the lysosomal compartment for degradation. The recruitment of TBC1D9B (red) or other TBC family GAP by the ATG8 homologues (yellow)  following autophagy signaling could inactivate the specific RAB, such as RAB11A as in the case of TBC1D9B recruitment, causing the conversion of the GTP status to the GDP status, and the detachment of the RAB and/or the effector molecule from the autophagosomes. This activity may thus prevent the latter to move to the recycling pathway, and allow them to go to the lysosomal compartment for fusion and degradation, thus promoting autophagic flux.

Multiple fusions with other types of vesicles could be quite common during the biogenesis/maturation of autophagosomes in mammalian cells. This process may very likely result in the recruitment of RAB molecules that are specific to the donor vesicles. Vesicles carrying different RAB molecules can become part of the autophagosomal membrane. However, continuous presence of these RAB molecules may “misguide” the autophagosome to the trafficking pathway specified by these RAB molecules and associated effector molecules, thus negatively affecting autophagosome development. We speculate that TBC1D9B is recruited to the autophagosomes to perform this inactivation function to avoid mis-trafficking (Fig. 7). Recruitment of TBC1D9B via LC3 can potentially avoid the autophagosome to be trapped in the recycling pathway specified by RAB11 and the associated effector molecules, allowing it to travel to the lysosomal compartment. Thus the presence of TBC1D9B would favor the autophagic flux and autophagic degradation.

This is certainly not the only way by which TBC family molecules may regulate autophagy. Notably, TBC1D14 can bind to activated RAB11 but has no GAP activity toward it^39^. Yet over-expressing TBC1D14 inhibited autophagosome formation by modulating the RAB11-positive recycling endosome, in addition to affecting trafficking pathway involving ATG9^39,44^. Among the other TBC family proteins that can affect autophagy and that can interact with the ATG8 homologues, TBC1D2A can serve as a GAP for RAB7^41,42^, which is important for its effects on autophagy^42,43^. TBC1D25 is recruited to autophagosomes to regulate RAB33B that interacts with ATG16L1^46^.

How GAP molecules are recruited to vesicles that carry the target RAB molecules is not entirely clear^40^. In the case of autophagy regulation, it seems that TBC family GAPs could be recruited through the interaction with the ATG8 homologues to the autophagosomal membrane. We found here that TBC1D9B can be located at the LC3-positive autophagosome compartment. Similarly, TBC1D5 was found to translocate from the endosome to the autophagosome under starvation treatment via its interaction domain with LC3^31^. TBC1D25 is also found to be recruited to autophagosomes by direct binding to ATG8 family members^46^. Thus interaction with ATG8 homologues can be a general mechanism for the recruitment of TBC family GAPs.

This hypothesis of how TBC1D9B may regulate autophagy indicates the complexity of mammalian autophagosome biogenesis. It is anticipated that other TBC family GAPs could regulate autophagy in this manner. Further studies would likely reveal new and detailed molecular mechanisms.

## Methods

### Screening for interacting protein of ATG8 homologues

Yeast two-hybrid screening was performed using the MATCHMAKER Two-Hybrid System (Clontech Laboratories, Inc. Catalog # K1609-1, Mountain View, CA). The open reading frame of human LC3B was inserted into the pLexA vector, which was transformed into yeast cells (strain EGY48). The transformed cells were not auto-activated on SD/-His-Ura+X-gal medium containing galactose and raffinose. The pLexA-LC3B-transformed cells were mated with the yeast strain YM4271 that contained a human brain cDNA library cloned on the pB42AD vector. The yeast cells were cultured on the YPD medium. Screening for colonies with interacting partners was performed on SD plates deficient in Leu, His, Trp and Ura (SD/-Leu-His-Trp-Ura), but supplemented with galactose, raffinose pentahydrate, and X-gal, known as the induced condition, for one week at 30 °C.

Quantitative β-galactosidase activity assay was performed as previously described^47^. Briefly, transformed yeast cells were plated on SD/Glu/-His/-Trp/-Ura plates for 3–5 days. Five colonies were selected to be plated on SD/Gal/Raf/-His/-Leu/-Trp/-Ura and X-gal plates at 30 °C for 2–4 days. The blue colonies were then transferred to the liquid SD/-His-Trp-Ura medium for overnight. The yeast cell density was measured by a spectrometer at A_600_, whereas the β-galactosidase activity was determined based on the level of o-nitrophenol at A_420_. The activity in *Miller Unit* is defined as (A_420(sample)_ − A_420(blank)_/(A_600_ × t × V), in which t is the reaction time in minutes, V is the volume of culture in milliliters.

To recover the cDNA of interacting proteins, the yeast DNA was extracted and the cDNA inserts were amplified by PCR with the following primers: forward 5′-ccagcctcttgctgagtggagatg-3′; and reverse 5′-caacaacgtatctaccaacgatttgaccc-3′, and identified by sequencing of the PCR products.

The pB42AD-inserts were rescued in *E*.*coli* (strain KC8). The interaction was confirmed by co-transforming pLexA-LC3B and the identified pB42AD-inserts into yeast strain EGY48, which were then cultured in the induced condition (SD/-Leu-His-Trp-Ura, supplemented with galactose, raffinose pentahydrate and X-gal) for one week at 30 °C. Positive colonies were identified by the blue color. Culture can also be maintained in non-induced condition on SD plate deficient in His, Trp and Ura, but supplemented with Glucose. This procedure was also used to examine the ability of mutant LC3B and TBC1D9B to interact with each other.

### Cloning and site-directed mutagenesis

Human LC3B, LC3A, GABARAP, GATE-16, ATG8L, TBC1D9Bα were amplified by RT-PCR from cDNA library made from HEK293. For the yeast two-hybrid system, LC3B, LC3A, GABARAP, GATE-16, or ATG8L was inserted into pLexA vector, whereas TBC1D9Bα and its mutants were inserted into pB42AD vector (Clontech Laboratories, Inc. Mountain View, CA). For expression in the mammalian cells, constructs were cloned into pEGFP-C2 or pcDNA3.1-FLAG vector. For preparation of GST-fused recombinant proteins, DNA inserts were cloned into pGEX-6p vector. For His-LC3B, LC3B was cloned into pET28a. All subcloned constructs were confirmed through DNA sequencing.

Site-directed mutagenesis was performed using QuikChange Lightning Site-Directed Mutagenesis Kit (Agilent Technologies, Santa Clara, CA) following manufacturer’s instruction. The construct was confirmed through sequencing.

### Preparation of recombinant proteins

To prepare the recombinant proteins DNA constructs in pGEX-6p vector or in pET28 vector were transformed into the BL21 strain of *E*.*coli*, which was cultured in LB medium. The bacteria were harvested and lysed with sonication in PBS (140 mM NaCl, 2.7 mM KCl, 10 mM NaHPO_4_, 1.8 mM KH_2_PO_4_, pH 7.3) containing protease inhibitors. To prepare GST-fused proteins, the lysate was incubated with glutathione-sepharose beads (Amersham Pharmacia Biotech, Little Chalfont, U. K.) at room temperature for 30 min. The recombinant protein was eluted with glutathione elute buffer (50 mM Tris-HCl, 10 mM reduced glutathione, pH 8.0) after the beads were washed with a 10x volume of PBS for three times. To prepare His-tagged proteins Ni-NTA resin (USB Corp., Cleveland, OH) was used to isolate the protein, which was eluted with 250 mM imidazole (pH 5.0) after the wash with PBS.

FLAG-TBC1D9B recombinant protein was prepared from HEK293 cells after the transfection of the construct in pcDNA3.1 vector and a stable cell line was selected. The stable cells were cultured in large-scale and cells were lysed in lysis buffer (50 mM Tris-HCl, 150 mM NaCl, 1% Triton X-100, and a protease inhibitor cocktail). The lysates were incubated with equilibrated anti-FLAG M2 antibody-coated beads for 1 hours at room temperature. FLAG-TBC1D9B was eluted with 0.1 M glycine HCl (pH 3.0), neutralized with 1/10 volume of neutralization buffer (0.5 M Tris-HCl, 1.5 M NaCl, pH 7.4).

### *In vitro* GST pull down assay

GST-fused recombinant proteins were incubated with glutathion-sepharose beads in equilibration buffer (20 mM Tris, pH 7.4, 0.1 mM EDTA and 100 mM NaCl) at 4 °C for 1 hour. The protein-coated beads were washed 4 times with equilibration buffer, and then incubated with cell lysates or recombinant proteins (FLAG-TBC1D9B, or His-LC3B protein) at 4 °C for 1 hour with gentle shaking. The beads were washed four times with equilibration buffer. The bound proteins were eluted by boiling the beads in sample buffer (125 mM Tris-HCl, pH6.8, 4% SDS, 20% (v/v) glycerol and 0.004% bromphenol blue), and resolved in SDS-PAGE, followed by staining with Coomassie Brilliant Blue (CBB) or by immunoblotting assay.

### Cell culture, transfection and siRNA-mediated knockdown

HEK293 cells were cultured in DMEM. A549 cells were maintained in RPMI1640. HCT116 cells were maintained in McCoy’s 5A. All media were supplemented with 10% fetal bovine serum and standard supplements. Cells were cultured in a humidified air atmosphere with 5% (v/v) of CO_2_ at 37 °C. For starvation treatment, cells were washed with phosphate-buffered saline (PBS) once and incubated in EBSS.

To establish stable cell lines, FLAG-TBC1D9Bα were introduced into HEK293 cells using Lipofectamine 2000 (Life Technologies, Inc. Waltham, MA) and cells were selected under G418 treatment. Alternatively, A549 stably expressing mCherry-TBC1D9B or GFP-LC3B were established using a lentivirus-mediated transfection Gateway system (Life Technologies, Inc.), and cells were selected under blasticidin treatment. The recombinant lentivirus was prepared in HEK293 cells.

The siRNA oligonucleotides targeting TBC1D9Bα were ordered from Qiagen (Hilden, Germany). The corresponding positions in the open reading frame (Fig. 6C) are: 813–833 (siRNA-1), 456–476 (siRNA-2), 519–539 (siRNA-3), and 1039–1059 (siRNA-4). The sequence of the scramble control siRNA is GGACGGAGCAGTACAA. These siRNA oligonucleotides were transfected into HEK293 using Lipofectamine 2000 for 72 hours. A549 cells with stable knockdown of TBC1D9Bα expression were generated using a pSIH-H1 lentivirus-mediated system. The scrambled control siRNA, shRNA-1, or shRNA-4 oligonucleotides were cloned into pSIH-H1-coGFP vector, which was then co-transfected with psPAX2 and pCMV-VSVG to HEK293ft cells to generate a recombinant lentivirus. A549 infected with the recombinant lentiviruses were selected based on the expression of coGFP in cells.

The effectiveness of siRNA-mediated knockdown was determined by RT-PCR. Total RNA was isolated from cells transfected with different siRNA using the RNeasy mini kit (Qiagen), and was converted to cDNA. Primers for TBC1D9Bα were 5′-gaccagctctcggactatc-3′ (forward) and 5′-cattgcagggagcccgtgct-3 (reverse). The PCR reaction was conducted at standard condition for 28 cycles. β-actin gene was amplified as a control.

### Immunoassay and preparation of polyclonal anti-TBC1D9B antibody

Cell lysates were prepared with 1% NP-40 lysis buffer with a cocktail of protease inhibitors (Roche Diagnostics GmbH, Mannheim, Germany). Protein concentrations were determined with the BCA protein assay kit (Pierce Biotechnology, Rockford, IL). For immunoblotting assay, proteins were resolved in standard SDS-PAGE, followed by transferring to PVDF membranes. The membranes were blocked in TBS containing 0.05% Tween 20 and 5% non-fat milk, incubated with primary antibodies, washed and incubated with a HRP-conjugated secondary antibody. The blots were developed with Western Lightning® Plus-ECL solution (PerkinElmer, Waltham, MA) and visualized with X-ray film. Intensities of the bands were quantified by Bio-Rad Quantity One® software (Bio-Rad, Hercules, CA). The densitometry ratios for treated samples versus the controls were determined for three biological replicates, which were normalization to the level of β-actin or GAPDH.

For co-immunoprecipitation assay, the lysate supernatant was incubated with EZview Red Anti-FLAG M2 Affinity Gel (Sigma-Aldrich, Cat. No. F2426, St. Louis, MO) overnight; the beads were washed 4–5 times with the lysis buffer. Immunoprecipitates were eluded by boiling the beads in 2× SDS loading buffer at for 5 min. The samples were subjected to Immunoblotting assay.

For polyclonal anti-TBC1D9B antibodies, we used two online antigenicity prediction algorithms, Kolaskar and Tongaonkar Antigenicity and Bepipred Linear Epitope Prediction, to define the sequence TEEDEPPAPELHQDAARELQ (AA^1081–1100^) of TBC1D9Bα as a peptide sequence with a high antigeneity. A peptide based on this sequence was synthesized (CHI Scientific, Maynard, MA) and used for the production of a rabbit polyclonal antibody (Cocalico Biologicals, Reamstown, PA). The antibody quality was examined against HEK293 expressing full-length GFP-TBC1D9Bα, or recombinant GST-fused TBC1D9B fragment containing the antigenic sequence. The production was approved by IACUC of University of Pittsburgh.

### Long-lived protein assay

Long-lived protein degradation assay was performed as previously described^48^. Briefly, cells were cultured in DMEM in 24-well plates. L-[^14^C]-valine was added to a final concentration of 0.2 µCi/ml to label intracellular proteins. Cells were labeled for 18 hours, followed by change to fresh medium with 10% cold L-valine for 1 hour to deplete labeled short-lived proteins. The cells were then incubated in EBSS or DMEM (plus 0.1% of BSA and 10 mM valine) with or without the other chemicals for an additional 6 or 16 hours. The culture medium was then recovered, from which the degraded long-lived proteins were measured via liquid scintillation (Beckman LS 6000).

### Statistical analysis

Statistical analysis was performed using GraphPad Prism v7.0. Values were expressed as mean ± SEM. One-way or two-way ANOVA followed by Tukey’s multiple comparisons test was used to assess significance. Significance level was set at p<0.05.
